# RADReef: A global Holocene Reef Rate of Accretion Dataset

**DOI:** 10.1038/s41597-024-03228-w

**Published:** 2024-04-18

**Authors:** Michael G. Hynes, Aaron O’Dea, Jody M. Webster, Willem Renema

**Affiliations:** 1 Naturalis Biodiversity Center, PO Box 9517, 2300RA Leiden, The Netherlands; 2 Institute for Biodiversity and Ecosystem Dynamics (IBED), Universiteit van Amsterdam, P.O. Box 94240, 1090GE Amsterdam, The Netherlands; 3 Smithsonian Tropical Research Institute, Box 0843-03092, Balboa, Republic of Panama; 4 Sistema Nacional de Investigación, SENACYT, Clayton, Republic of Panama; 5 Geocoastal Research Group, School of Geosciences, The University of Sydney, Sydney, NSW 2006 Australia

**Keywords:** Geomorphology, Physical oceanography, Palaeoceanography, Biogeochemistry

## Abstract

Reef cores are a powerful tool for investigating temporal changes in reef communities. Radiometric dating facilitates the determination of vertical accretion rates, which has allowed for examination of local-regional controlling factors, such as subsidence and sea level changes. Coral reefs must grow at sufficient rates to keep up with sea level rise, or risk ‘drowning.’ As sea level is expected to rise significantly in the next 100 years and beyond, it is important to understand whether reefs will be able to survive. Historical records of reef accretion rates extracted from cores provide valuable insights into extrinsic controlling factors of reef growth and are instrumental in helping predict if future reefs can accrete at rates needed to overcome predicted sea level changes. While extensive research exists at local and regional scales, limited attention has been given to identifying global patterns and drivers. To address this, we present “RADReef”: A global dataset of dated Holocene reef cores. RADReef serves as a foundation for further research on past, present and future reef accretion.

## Background & Summary

Most coral reefs in the tropics and subtropics have been accreting vertically at varying rates throughout the Holocene, resulting in thick sequences of carbonate build-up that provide a unique habitat for scores of biodiversity and delivering enormous ecological, sociological and economic benefits across the globe^1^. To preserve biodiversity and sustain ecosystem services, reefs must continue to accrete, yet it is unclear if these rates will be sufficient to prevent drowning under predicted scenarios of future sea level and temperature change, especially if local stressors continue to reduce the capacity of reefs to net accrete in the face of bioerosion^2–4^.

One approach is to use dynamic net accretion models (or carbonate budgets) to predict how reefs will respond under future projections of local sea level rise^4–7^. However, these models require a complex understanding of local biotic and abiotic conditions (such as carbonate production, erosion, and more) limiting their application over larger spatial scales. An alternative approach is to observe how reef accretion has fluctuated in the past by using reef matrix cores^7–12^, thereby intrinsically accounting for local conditions. Several studies have demonstrated the potential applications of dated reef cores to predict a reef’s potential to accrete^13–18^, but these studies remain limited in geographic scope. Many reefs have been cored and dated globally, but so far, a standardized compilation of global/regional Holocene reef accretion has not been produced. Such data could offer new tools to explore the controls on reef accretion and help make general and local predictions in the face of future change.

In this study we present a global Reef Rate of Accretion Dataset (RADReef) from published, radiometrically dated Holocene reef cores from around the world. The aim of the dataset is to enable a spatio-temporal framework of reef accretion data from which questions about the past drivers and future predictions of reef accretion can be explored more in depth. Here we review the quality and range of the data assembled and show examples of how this dataset could be potentially useful in future research.

## Methods

### Study selection

To build RADReef we compiled radiometric dates and depths in fossil coral reef cores from peer-reviewed published studies. This was achieved by searching on Google Scholar (https://scholar.google.com/) for “reef cores” and then selecting studies that meet the minimum criteria of a study containing at least one core with two or more radiometric dates from different depths made using radiocarbon (^14^C) or Uranium-Thorium (U-Th) radiometric dating approaches on carbonate producing organisms^19–21^. Carbonate producing organisms generally consisted of corals and marine mollusks, and lesser so foraminifers. This minimum requirement was to allow for the calculation of a Mean Accretion Rate (MAR). For RADReef, dates were restricted specifically to the Holocene. Fifty-seven cores also included Pleistocene dates. These were included in RADReef but were not used to estimate MAR’s. In total we compiled data from 117 studies, comprising a total 1,066 cores, with 3,535 dates, from 162 localities. Here a locality is defined as the reported designation of a coring site from a study.

### Metadata

**Fig. 1:**
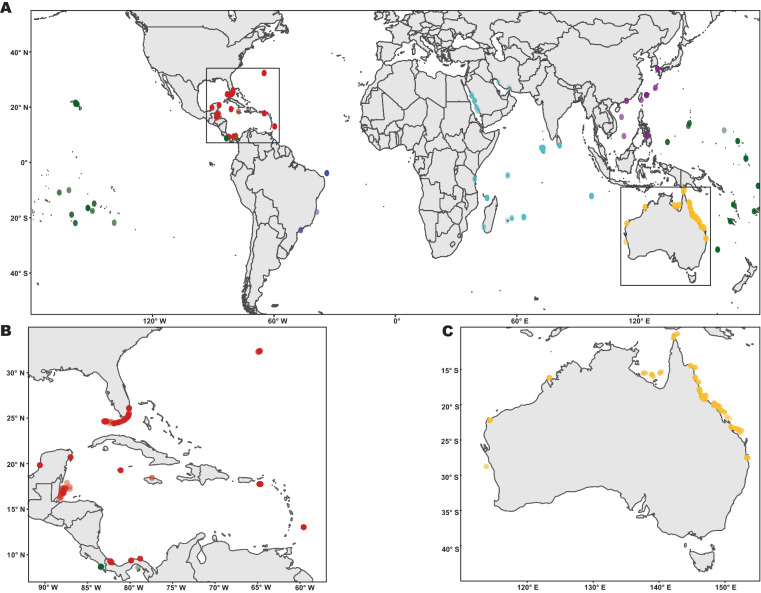
(**A**) Map of core locations used in RADReef globally. Colors correspond to regions. (**B**) Map of core locations in the Caribbean and Tropical Eastern Pacific. (**C**) Map of core locations in Australia.

The depth of the seafloor at each coring site was compiled when reported by the original study, otherwise measured from figures presented in the study, if available. If neither of these were available, then depth was not determined and therefore paleoreef depth (PRD) could not be calculated. PRD is the sum of the present day water depth (water depth at the time of core collection) combined with the depth of a dated point within the same core^22^. This allowed for direct comparisons of depths from different environments/water levels. Latitude and longitude data (in decimal degrees format) were compiled or extracted from figures showing the coring locations when not provided numerically. For regional comparison, data was separated into regions which were chosen by a concentration of available cores comparable to paleo sea level regions^23^ (Fig. 1). The Southwest Atlantic region was represented by only 10 cores, with no dates older than 5700 YBP, and therefore this data was only used for global calculations.

### Compilation and adjustment of radiometric dates

All studies that provided uncalibrated radiocarbon dates were re-calibrated using CALIB version 8.1.0 and the Marine20 calibration curve^24,25^ and reported in years before present (YBP), with present being AD1950^26^. This uniform approach created uniformity of data for comparison across reefs and regions. Local radiocarbon reservoir correction (ΔR) values were taken from within the Marine20 Reservoir Database^24,25^. When possible, the ΔR was taken directly from a locality, otherwise the nearest ten points from the reservoir database were averaged to determine the ΔR value. Calibrated ^14^C (without the accompanying uncalibrated data) and U-Th dates were left unadjusted but were converted to YBP where necessary.

### Accretion rate calculations

**Fig. 2:**
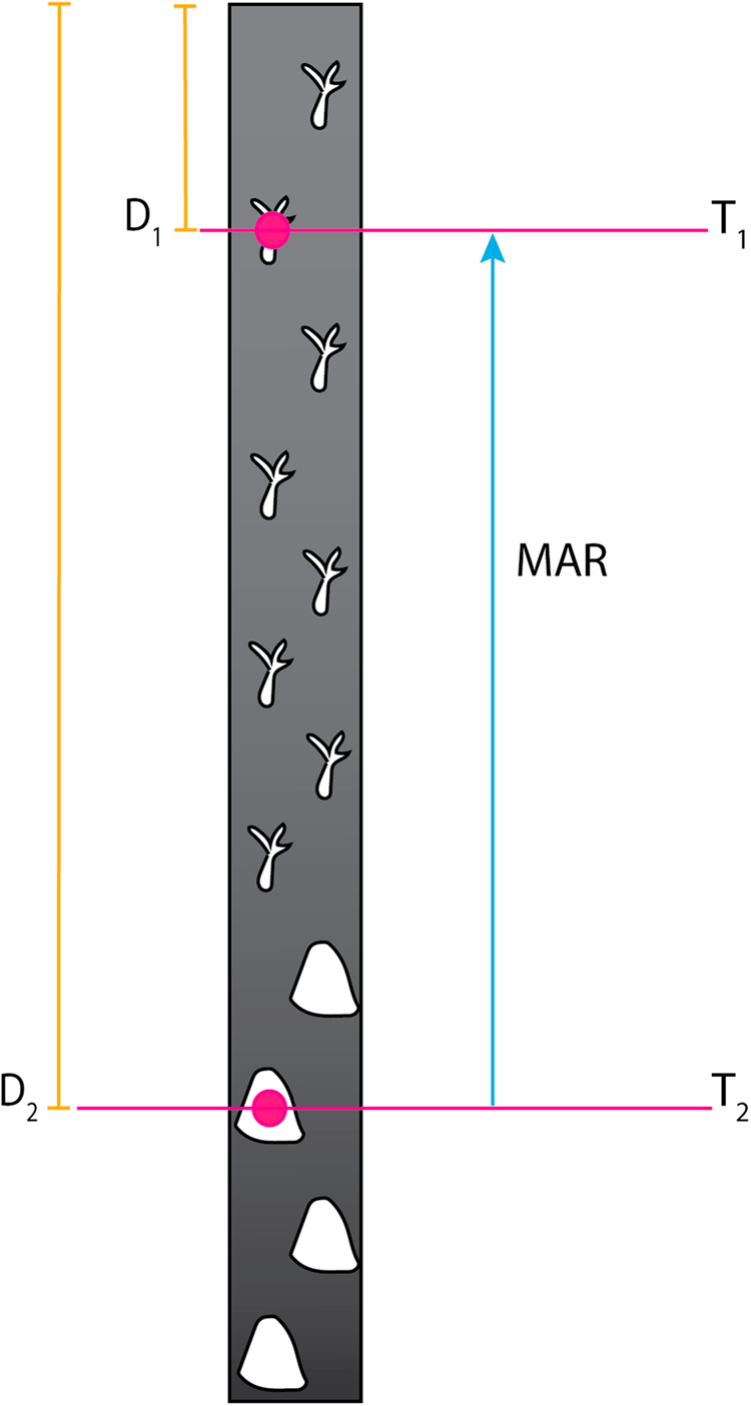
A diagram of a core with two dated points to demonstrate how MAR and Mean Age are calculated.

**Fig. 3:**
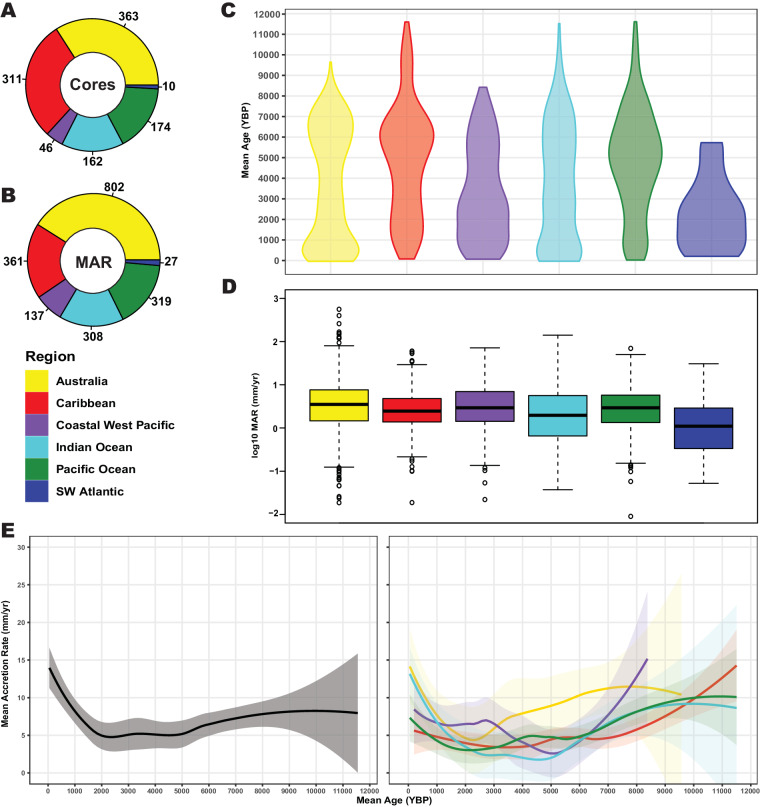
(**A**) The number of cores included in RADReef shown by region. (**B**) The number of MAR in RADReef shown by region. (**C**) Violin plots showing the distribution of Mean Ages in RADReef by region. (**D**) Boxplots of MAR (log10 transformed) by region. (**E**) MAR vs Mean Age curves of the Holocene showing changes in MAR through time. Grey curve is Global, colored curves are regions.

**Fig. 4:**
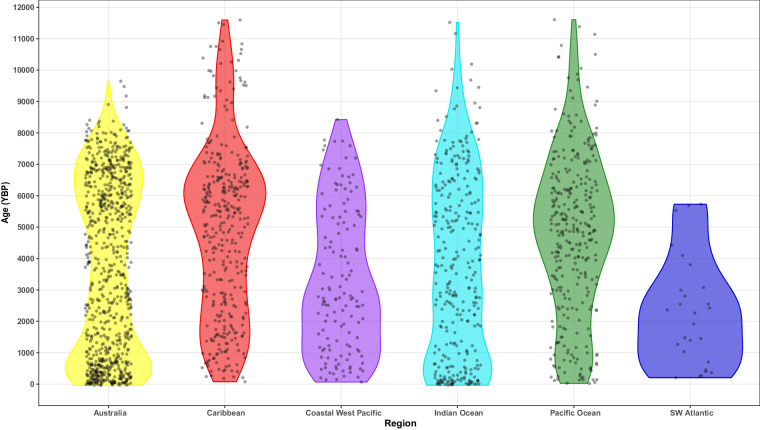
Violin plots of Mean Ages by region in RADReef with data points included.

**Fig. 5:**
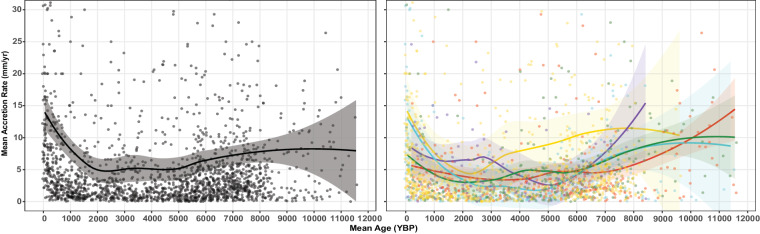
MAR curves globally and regionally respectively, with data points included.

MARs were calculated using the formula *MAR* = (*D*_2_-*D*_1_)/(*t*_2_-*t*_1_), where *D* is the depth within the core, and *t* is the dated age of the core at that depth (Fig. 2). Negative accretion rates represented age reversals within the core and were excluded. When replicate dates were available from the same depth in one core, we chose the date with the lowest dating error (σ) that did not cause an age reversal to calculate MAR. MARs were also log_10_ transformed to facilitate regional comparison box plots (Figs. 3D). Mean time (Mt) was determined by calculating the mean of *t*_*1*_ and *t*_*2*_ used in the MAR formula (Figs. 3C, 4). MAR was plotted against its respective Mt to generate accretion rate curves, using a locally estimated scatterplot smoothing (LOESS) regression models with 95% confidence intervals (CI) on the Mean Ages and MAR (Figs. 3E, 5). Accretion rate curves were generated for each region except the Southwest Atlantic region where n was too low, although data was included in the global accretion rate curve.

## Data Records

RADReef is currently housed in figshare^27^ as three separate comma separated values (csv) files (10.6084/m9.figshare.25251157). The first file RADReef READ ME, is a description of the metadata categories contained within RADReef. The actual dataset csv file, RADReef, is where all the data for each dated point is available, including calculated MAR’s (Table 1), metadata, and the reference (ordered numerically in the csv files) for the original data source^7,8,10,12–14,19,28–136^. Finally, RADReef References is a csv sheet containing the full citation for the original data linked to the number recorded in RADReef. Additionally, we have broken up the RADReef data file into individual csv files by region and by reef environment for an ease-of-use practice for users.

There are differences in the number of cores per region with over half of the cores from the Australian (336 cores) and Caribbean regions (209 cores), followed by one third from the Indian Ocean (168 cores) and Pacific Ocean (180 cores) regions. The Coastal West Pacific region (46), is an area from which more core data is needed to improve the robustness of RADReef, (Fig. 1A and Table 2). The data deficient Southwest Atlantic region (10) could also benefit from a more comprehensive core dataset, but is hindered by the low number of reefs in the region.

**Table 1:** Mean Accretion Rates (MAR) regionally and globally, separated into statistical categories.

Region	Min MAR (mm/yr)	Max MAR (mm/yr)	Mean MAR (mm/yr)	Median MAR (mm/yr)	SD MAR	Variance MAR
Australia	0.018644	560	9.51718	3.52403	30.24483	914.74951
Caribbean	0.018747	60	4.89246	2.46154	7.828564	61.28642
Coastal West Pacific	0.022044	71.5	5.97996	2.92683	9.81349	96.30459
Indian Ocean	0.037113	140.5882	6.33054	1.96721	14.52312	210.92122
Pacific Ocean	0.008993	69.56522	5.36127	2.94118	7.904625	62.48309
Southwest Atlantic	0.052257	30.66667	3.558681	1.09756	6.585944	43.37466
**GLOBAL**	**0.008993**	**560**	**7.140957**	**2.90452**	**21.00262**	**441.11**

**Table 2:** Metadata of core occurrences regionally and globally showing the spread of available data.

Region	# of Locations	# of Cores	# of Dates	# of MAR
Australia	61	363	1370	802
Caribbean	20	311	752	361
Coastal West Pacific	13	46	214	137
Indian Ocean	31	162	544	308
Pacific Ocean	34	174	610	319
Southwest Atlantic	3	10	45	27
**GLOBAL**	**162**	**1066**	**3535**	**1954**

Two thirds of the dates were obtained by radiocarbon analysis (2,225) and the other third using Uranium-Thorium methods (1,310). The number of dates per core varied greatly, with the mode of 1-2, a median of 2 and a mean of 3 (3.38, SD 3.13) dates per core (Table 3) (209 and 215 cores respectively). Thirty-two dates in a single core was the most dated core in RADReef. There were 155 cores with 6 or more dates, and another 92 cores with 5 dates, comprising about one quarter of all cores. These well-dated cores offer a thorough examination of reef accretion in a particular location. All regions, except the Indian Ocean and the Coastal West Pacific, have two dates per core as the most common occurrence in RADReef.

**Table 3:** Number of dates per individual core regionally and globally.

Region	1 Date	2 Dates	3 Dates	4 Dates	5 Dates	6+ Dates
Australia	57	80	58	48	31	62
Caribbean	44	48	37	29	23	28
Coastal West Pacific	5	6	8	12	6	9
Indian Ocean	64	35	12	17	12	28
Pacific Ocean	39	43	33	21	17	27
Southwest Atlantic	0	3	2	1	3	1
**GLOBAL**	**209**	**215**	**150**	**128**	**92**	**155**

There were a total of 1,954 independent MAR values in the RADReef dataset (Fig. 1B,D, Table 2). The total number of MARs from each region varies, and some regions are more represented than others. Australia has almost a third of all the MAR’s (802), while the Caribbean (361), Indian (308) and Pacific Ocean regions (319) were similarly represented. Other regions were less well represented. For example, the Coastal West Pacific region only produced 137 MARs despite being the center of coral diversity (the Coral Triangle). The Southwest Atlantic region only produced 27 MARs.

Globally MAR in RADReef had a mean MAR of 7.14 mm/yr (SD; 21 mm/yr; Table 1). We see in all regions except the Southwest Atlantic a maximum MAR of over 50 mm/yr (which is likely an artificial exaggeration of MAR due to compaction, bioturbation, and/or other factors). The minimum MAR for all regions in the global RADReef dataset is just above 0 mm/yr. The standard deviation for all regions, excluding Australia (30 mm/yr) and the Indian Ocean (14.5 mm/yr), are below 10 mm/yr. Australia sees a similarly high SD as the Global RADReef mean due to the largest number of MAR values for any region, including the maximum of 560 mm/yr, and is therefore driving the global patterns. The Indian Ocean also has a maximum MAR of 140 mm/yr which would also impact the mean MAR in the region. However, the mean MAR across all regions varies from 3.56–9.52 mm/yr with the Global RADReef MAR having a mean rate of 7.14 mm/yr (Table 1). The Median MAR varies between 1.098–3.524 mm/yr within regions and globally the median MAR is 7.141. These mean and median rates are all within the expected range of net vertical accretion/coral growth^5,6,31,137^ and gives confidence that RADReef data is a valuable and robust resource with which to study Holocene reef accretion.

## Technical Validation

The dataset was produced by maintaining a minimum selection criteria of individual studies as detailed in Methods. These were only selected if they were published articles or books from reputable sources, and no gray literature or privately stored data was used. A direct link to all studies included is available on the RADReef references csv, and is described as a DOI when possible, or other link when no DOI was available^27^. Further, all data underwent standardization and recalibration when possible, for consistency. Radiocarbon dates being recalibrated using ΔR values from the same database source aided in this consistency. PRD was used as a standardized depth comparison method for all data points examined. Finally, a standard formula for calculating MAR was used, based on using two points from a core, and was applied to the entire dataset. Some studies calculated accretion rate by simply dividing the core depth by the age to get a localized accretion rate, but by using the MAR formula a more accurate measurement of accretion between time points was gained. This is accompanied by a list of whether, and why, a MAR could not be obtained for certain data points within RADReef, and is noted on all csv files containing RADReef data. There were limitations on the data collection, which are discussed below .

## Usage Notes

RADReef is stored under a CC-BY 4.0 license on figshare (10.6084/m9.figshare.25251157) and therefore is subject to citation standards set forth by the license^27^. It should be noted that some published data could not be included in RADReef. The most common reason was that data did not meet the minimum criteria (see earlier) and a lack of standardization of reef core data collected. Reporting of metadata in core studies used variable units (feet or m) and different methods of measurements (core depth from sea floor, from Mean Sea Level, from Lowest Astronomical Tide). This was especially prevalent in the earliest core studies. Metadata, such as water depth, core depth, and specific location were not always numerically reported and had to be manually ascertained by the authors of RADReef from figures, thereby reducing their accuracy and direct comparability. Furthermore, some studies, especially the oldest ones, did not report the uncalibrated ^14^C dates, making it impossible to recalibrate using the latest edition of Calib and the Marine20 curve^25,138^. Nevertheless, the majority (93.5%) of ^14^C dates were recalibrated. RADReef records if a date could not be recalibrated, caused an age reversal issue, or any other uncertainty.

## Data Availability

No custom code was generated in this work.
